# Single molecule studies of dynamic platelet interactions with endothelial cells

**DOI:** 10.3389/fbioe.2024.1372807

**Published:** 2024-04-03

**Authors:** Fabian Hauser, Christoph Naderer, Eleni Priglinger, Anja Peterbauer, Michael B. Fischer, Heinz Redl, Jaroslaw Jacak

**Affiliations:** ^1^ Department of Medical Engineering and Applied Social Sciences, University of Applied Sciences Upper Austria, Linz, Austria; ^2^ Austrian Cluster for Tissue Regeneration, Vienna, Austria; ^3^ Department of Orthopaedics and Traumatology, Johannes Kepler University Linz, Linz, Austria; ^4^ Red Cross Blood Transfusion Service for Upper Austria, Linz, Austria; ^5^ Department for Biomedical Research, Center of Experimental Medicine, Danube University Krems, Krems, Austria; ^6^ Clinic for Blood Group Serology and Transfusion Medicine, Medical University of Vienna, Vienna, Austria; ^7^ Ludwig Boltzmann Institute for Traumatology in Cooperation with the AUVA, Vienna, Austria

**Keywords:** platelets, microfluidics, endothelial, vessel-on-a-chip, single molecule localization microscopy, super-resolution microscopy, machine learning

## Abstract

A biotechnological platform consisting of two-color 3D super-resolution readout and a microfluidic system was developed to investigate platelet interaction with a layer of perfused endothelial cells under flow conditions. Platelet activation has been confirmed via CD62P clustering on the membrane and mitochondrial morphology of ECs at the single cell level were examined using 3D two-color single-molecule localization microscopy and classified applying machine learning. To compare binding of activated platelets to intact or stressed ECs, a femtosecond laser was used to induced damage to single ECs within the perfused endothelial layer. We observed that activated platelets bound to the perfused ECs layer preferentially in the proximity to single stressed ECs. Platelets activated under flow were ∼6 times larger compared to activated ones under static conditions. The CD62P expression indicated more CD62P proteins on membrane of dynamically activated platelets, with a tendency to higher densities at the platelet/EC interface. Platelets activated under static conditions showed a less pronounced CD62P top/bottom asymmetry. The clustering of CD62P in the platelet membrane differs depending on the activation conditions. Our results confirm that nanoscopic analysis using two-color 3D super-resolution technology can be used to assess platelet interaction with a stressed endothelium under dynamic conditions.

## 1 Introduction

Two-color 3D single molecule localization microscopy (SMLM) found its way to be a key technology in the analysis of single cells via 3D nanoscopy. Correlation of data from simultaneously acquired 2-colour SMLM images is often required, with one color encoding relevant nanoscopic information and the second providing a super-resolution image of larger, diffraction-limited objects. Here, nanoimaging adds value to the consensus of data analysis by providing a larger parameter space for machine learning-based image processing to determine spatial orientation of cell organelles (e.g., Mitochondria). One of the current topic where SMLM analysis has proven relevant over the last few years is quantification of platelet activation and thrombus formation (Knight et al., 2017; Bergstrand et al., 2019; Mayr et al., 2020; Chung et al., 2021; Go et al., 2021). For platelets (with a physiological size of 1–3 µm in the circulation and a diameter 5–8 µm when spread following activation (Zarka et al., 2019)), access to information at the nanoscopic level is key to a better understanding of cellular processes related to blood vessel remodeling, for e.g., aggregation due to clot formation (Fogelson et al., 2012; Brouns et al., 2020; Scavone et al., 2020). In order to create a physiologically relevant parameter space, flow conditions in the blood vessels need to be imitated. *In vitro* modelling of blood vessels under flow conditions has been performed for quantifying platelet adhesion (Tsai et al., 2012; Jain et al., 2016; Brass et al., 2019; Oshinowo et al., 2020; Buchegger et al., 2021; Dupuy et al., 2021). Platelet adhesion to intact endothelium is inhibited by the glycocalyx (Vink et al., 2000) and mediators released by endothelial cells (e.g., fibronectin or von Willebrand Factor) (Neubauer and Zieger, 2021). Stressed endothelium, however, can expose pro-thrombotic surfaces to initiate platelet adhesion, where the glycoprotein (GP)Ib-IX-V receptor binds to von Willebrand Factor protein. When individual platelets are activated, additional platelets from the bloodstream can adhere and promote aggregation. Platelet aggregation is mediated by the interaction of the CD41/CD61 complex (GPIIb/IIIa) with fibrinogen (Sebastian and Dittrich, 2018) or the binding of extracellular matrix components such as collagen, laminin and fibronectin to GPVI. Signals via these GPs can depend on shear stress which affects platelet morphology and function. At low shear stress values (<4 Pa), platelets are spherical shaped and can form filopodia; at higher values, platelets are more discoid shaped and can form additional tethers (Jackson, 2007). All these factors influence platelet function.

Cellular stress on the EC layer can disturb haemostasis and can trigger platelet activation facilitating platelet binding to the endothelium (Yau et al., 2015). Stressed ECs can produce reactive oxygen species, and while the damaging effects of reactive oxygen species is well determined, a recent paradigm shift has shown that mitochondrial reactive oxygen species can also act as signaling molecules to activate pro-growth responses in ECs. One possibility to assess cellular stress in ECs is to investigate changes in mitochondrial network formation. Mitochondria constantly undergo fusion into highly branched networks and fission into smaller punctate and rod-like structures (Valente et al., 2019). Long mitochondria networks indicate healthy and stress-resistant ECs; a high number of short networks or punctate indicate the presence of cellular stressors causing damage or enhanced regeneration according to the intensity of the stress signal (Eisner et al., 2018). Quantitative diffraction limited imaging of the mitochondrial morphology enables 2D and 3D classification of cellular health and disease (Valente et al., 2017; Harwig et al., 2018; Zahedi et al., 2018; Chaudhry et al., 2020; Chu et al., 2022). The parameters of platelet activation that correlate with the distribution of mitochondria in endothelial cells, are an indicator of their physiological state and function.

In this work, we apply two-color 3D localization microscopy, implemented in a customized microfluidic system with primary human CD34^+^ umbilical cord blood cells (Pedroso et al., 2011; Cecchelli et al., 2014) for simultaneous quantitative nanoscopic analysis of platelet activation on variously stressed *in vitro* adopted ECs, as in a model system. For this purpose, the spatial distribution of the platelet activation marker CD62P (P-selectin) was determined and compared with the mitochondrial distribution in ECs within the perfused layer. In our model system, a confluent EC layer in the microfluidic was perfused until more than 65% of the ECs were flow-oriented (most of them are predominantly oriented in the center of the channel). A femtosecond laser was focused onto single ECs to selectively inflict cellular stress on individual ECs within the orientated, confluent cell layer. These laser-treated ECs (ltECs) complemented the naturally occurring population of stress-resistant ECs (srECs) and naturally stress-prone ECs (spECs, due to cultivation and handling). The two-color 3D SMLM imaging provided a 3D localization map of mitochondrial distribution within the ECs, enabled us to classify the ECs into three groups and spatially correlate them to CD62P distribution in platelets. The spECs and ltECs showed mitochondrial network fragmentation, while srECs maintained their typically long and branched mitochondrial networks. The 3D SMLM localization maps of immune-fluorescently labelled mitochondria were used to assess the mitochondrial network integrity using a custom software tool for the segmentation of puncta, rod, and network structures. This enabled the classification of ECs based on their mitochondrial morphology: srECs, spECs and ltECs. Simultaneously, we quantified anti-CD62P (P-selectin) density in the second color channel of 3D SMLM images to monitor platelet activation and to observe platelet reaction to ECs with different stress levels. Our 3D SMLM data of CD62P signals enabled a quantitative comparison of platelet volumes and densities under static or flow conditions.

## 2 Materials and methods

### 2.1 Microfluidics fabrication

A 175 µm thick PET foil (Optimont 501, Bleher Folientechnik GmbH) was sandwiched between two double-sided adhesive tapes (Adhesive Research Arcare 90445, thickness 80 µm) for a total thickness of 335 µm. A single straight microfluidic channel (36 × 1.5 mm) was cut from this foil using a craft cutter (Silhouette Portrait 2) (Kokalj et al., 2014). The top was sealed by an impermeable 30 µm thick PET foil (Bleher Folientechnik GmbH). Two tube connectors (EV Group) were fixed on top using double-sided adhesive tape. The bottom layer was a microscope coverslip (Menzel 24 mm × 50 mm, #1 SPEZIAL, Thermo Scientific). The coverslip enabled observation of the sample area with a high magnification/high NA objective lens. To give the chip the required rigidity, a 3 mm acrylic glass frame was fixed on top using double-sided adhesive tape. After assembly, the microfluidic chip was baked at 60 C for 1 h. Tubing and the microfluidics chip were placed into a petri dish and properly sealed using parafilm for UV sterilization. Each side was exposed to UV light for 2 min (Dymax ECE, United StatesA). Tubing, tube connectors, and acrylic frame were reused after proper washing with 1% sodium dodecyl sulfate, 70% 2-propanol and deionized water.

### 2.2 Automated cell counting using deep learning

A convolution neuronal network (CNN) was trained for the automatic segmentation of cell nuclei. The centers of nuclei were indicated by normalized 2D symmetrical Gaussian functions with a sigma of 5 pixels. The network was trained using phase-contrast images of cultivated ECs in which each nuclei center was labelled by hand (N = 364). Images with varying EC densities and illumination settings were acquired. A standard inverted microscope (Axiovert 135, Carl Zeiss) using a 10x air phase contrast objective lens and microscopy camera (AxioCam MRc5, Carl Zeiss) was used. The architecture of our CNN is based on the residual neuronal network (He et al., 2016) combined with a U-Net (Xie et al., 2018; Falk et al., 2019) (4 layers deep, Supplementary Figure S3). Full-size grayscale phase contrast images (1,292 × 968 pixels, 0.68 µm pixel size) and target images containing the nuclei centers were cut into smaller 128 × 128 sub-images with 50% overlap. The training was performed on a NVidia RTX 3060 graphics card (12 GB VRAM) using the Keras (TensorFlow v2.10.1) (Chollet, 2015) backend, binary cross entropy loss function and ADAM optimizer (Kingma and Ba, 2015). Our CNN converged after about 9 epochs (batch size: 128, training images: 45,360, validation images 5,040). Full-size images (1,292 × 968 pixels) were reconstructed from overlapping smooth blended predicted images (128 × 128 pixels). We used a second-order spline window function for blending with 50% overlap (Chevalier, 2017). Positions of nuclei centers from predicted full-size images were determined by non-maximum suppression (Neubeck and Van Gool, 2006) (window size: 19 pixels) of the predicted Gaussian centers. Peak values also showed how certain the network was about detected cell nuclei. Based on this CNN we build a graphical user interface which allows the user to approximate the cell density during cultivation without detaching the cells. The approximate cell count can be calculated by multiplying the density with the area of the used cell culture dish.

### 2.3 Cultivation of EC

Primary human ECs were differentiated from CD34^+^ cells isolated from human cord blood (Pedroso et al., 2011; Cecchelli et al., 2014) and were provided in frozen aliquots of 10^6^ cells at passage five by Prof. Fabien Gosselet, Université d’Artois, France. After thawing, cells were seeded onto gelatine (0.1% in PBS)-coated 10 cm-dishes (Treated, 100 × 20 mm, Corning) in ECM-5 (ECM, Sciencell) supplemented with 1% endothelial cell growth supplement (Sciencell), gentamycin (50 μg/mL, Biochrom AG, ref A-2712), and 5% of preselected, heat-inactivated FBS and cultivated at 37 C, 5% CO2. After reaching confluency, ECs were washed 3 times with prewarmed PBS, detached with a trypsin/EDTA solution, counted, and seeded at approximately 5 × 10^5^ cells/mL. Expression of the EC marker CD31/PECAM-1 was confirmed by flow cytometry (data not shown) and immunofluorescence (Supplementary Figure S4).

The UV-sterilized microfluidic chip was coated using 0.1% gelatine solution for 15 min at 37 °C. Next, the tubing (1.52/3.22 mm inner/outer diameter respectively, Roth) with a length of 290 cm, a peristaltic pump (Ismatec, ISM930, 4 Channel) and long needles (Sterican^®^, 0.80 × 120 mm, B. Braun) which were inserted into a sterile bioreactor tube (Tubespin^®^ Bioreactor 50 with a septum, TPP, #86050) were attached to the microfluidic chip forming a closed loop. The tubing was filled up to the 3-way stopcock (Discofix^®^ 3SC, B. Braun) using prewarmed ECM-5. Suspended cells (1.6 × 10^5^ cells/mL) were transferred into the microfluidic chip via a septum connected to the perpendicular connector of the 3-way stopcock utilizing a 1 mL syringe (Omnifix®-F, B. Braun) with 0.70 × 30 mm needle (Sterican^®^, B. Braun). Next, the microfluidic chip and bioreactor tube containing the ECM-5 (5 mL) was placed into the incubator with tubing still connected to the peristaltic pump outside. The peristaltic pump was connected to a computer controlling the perfusion using custom-written software. For the first few days, the pump was active (119 μL/min) for 5 min every 4 h until confluence was reached. After confluence, the perfusion time was gradually increased (5 min, 10 min, 20 min, 30 min) whereas the delay time was stepwise decreased (3 h, 2 h, 1 h 0.5 h and 0.25 h) until constant flow was reached. Cells were kept under constant flow (up to 7 days). ECM-5 within the bioreactor reservoir was changed every 2 days. Every day, cell morphology was observed by an inverted microscope (Axiovert 135, Carl Zeiss). The EC density was determined using an automated cell counting CNN from acquired phase-contrast images (as described above). Experiments were performed, in case >65% of cells orientated into flow directions (after two to 7 days). EC tight junction formation was validated using fluorescent markers against CD144/VE-cadherin (see Supplementary Figure S1C,D) and CD31/PECAM-1 (see Supplementary Figure S4D-F).

ECs under static conditions were prepared as mentioned above. After confluence, ECs were washed 3 times with prewarmed PBS, detached from the 10 cm dish with trypsin/EDTA solution and counted. The middle 2 chambers of a Lab-Tek™ chambered cover glass (155382, 4 chambers, Nunc, Thermo Scientific) were coated with 0.1% gelatine solution for 15 min at 37 °C. ECs were seeded at approximately 3 × 10^5^ cells/cm^2^ and incubated in 900 µL ECM-5 for 4 days, the medium was exchanged every 24 h.

### 2.4 Human platelet concentrate

Single donor platelet concentrates were provided by the Red Cross Blood Transfusion Service (Linz, Upper Austria). All samples were collected during routine thrombocyte apheresis in accordance with the policies of the Red Cross Transfusion Service, Linz. All blood donors signed their informed consent that residual blood material can be used for research and development purposes. All experimental protocols were approved by and carried out in collaboration with the Red Cross Blood Transfusion Service, Linz.

Platelet concentrates were generated by single platelet apheresis using an automated cell separator (Trima Accel Automated Blood Collection System, TerumoBCT) at the Red Cross Blood Transfusion Service (Linz, Upper Austria). All blood donors signed an informed consent that blood material can be used for research and the study was conducted in accordance with the policies of the Red Cross Transfusion Service. Platelets were finally stored in SSP+ (Macopharma) and ACD-A (acid citrate dextrose + adenosine, Haemonetics^®^ anticoagulant citrate dextrose solution, Haemonetics^®^, Braintree) was used as an anticoagulant. 2 mL of the platelet concentrate (containing ∼1 × 10^6^ platelets/µL) were aseptically transferred into a separate storage bag and experiments were carried out within 24 h after donation.

### 2.5 Laser-induced cell injury

A Workshop of Photonics (WOP) multiphoton lithography instrument equipped with an ultrashort pulsed laser (CARBIDE, 1 MHz repetition rate, 290 fs pulse duration, Light Conversion) with two available wavelengths (1,030 nm and 515 nm) was used for cell treatment. The laser beam was focused with a 50× magnification air objective lens (NA = 0.42, Mitutoyo). A 3-axis stage (AEROTECH Nanopositioner) was used for sample motion.

The medium reservoir within the bioreactor tube was exchanged with a mixture of 10% human platelet concentrate and ECM-5. The whole microfluidic chip was taken out of the incubator. Since the microfluidic chip and tubing formed a closed system, no further precautions to keep a sterile environment were necessary. After the microfluidic chip was placed within the WOP imaging chamber, flow was applied (119 μL/min) which allowed the platelets to interact with the ECs during the laser treatment. Depending on the duration of the laser experiment (max 1 h), up to 5 single cells within the center of the channel were selected and subsequently, laser treated using the 515 nm laser at 25%–30% power (1.22–1.38 mW peak power in the focal plane). The ECs’ nuclei were focused during the laser treatment procedure until a visual change within the cell’s cytoplasm was observable or blebs formed (nuclei were treated for several seconds each). Each ltEC’s position was marked on both edges of the microfluidic channel using 100% laser power. The treatment time was limited to 1 h to limit the stress of all ECs due to the environmental change (temperature, gas). Afterwards, the microfluidic chip was put into the incubator at 37°C/5% CO2 for 15 min under flow conditions with platelets to recover.

### 2.6 Fluorescence microscopy

For immunostaining, cells were rinsed in pre-warmed HBSS containing Ca^2+^ and Mg^2+^. Cells were then fixed with 4% paraformaldehyde in cytoskeleton buffer with sucrose (CBS, according to a protocol of Louise Cramer (Symons and Mitchison, 1991)) for 20 min at room temperature. Cells were permeabilized in 0.5% Triton X-100 with CBS for 10 min and blocked in 10% albumin from chicken egg white (Sigma-Aldrich, Vienna, Austria) in CBS for 30–60 min. P-selectin (CD62P, 100 μg/mL, BioLegend) conjugated Alexa Fluor^©^ 647 was used to stain activated platelets. Mitochondria of cells were stained using an anti-mitochondria monoclonal antibody (500 μg/mL, Sigma-Aldrich) conjugated Alexa Fluor^©^ 488. For co-staining CD62P and mitochondria, a solution of 1:150 anti-CD62P and 1:100 anti-mitochondria antibody diluted in CBS was used to stain ECs and platelets for 1 h and washed 10 times using PBS.

Fluorescence microscopy images were acquired using a modified Olympus IX81 inverted epi-fluorescent microscope with an oil-immersion objective lens (PlanApo N, 60×, NA 1.42, Olympus). Samples were mounted on a XYZ piezo stage (PI Mars; P-562.3CD, Physical Instruments) which has nanometer accuracy, combined with a coarse mechanical stage with a travel range of 1 cm × 1 cm (Hybrid, JPK Instruments). A tube lens with an additional magnification of 1.6 was used to achieve a final imaging magnification of 96 (corresponding to a pixel size of 167 nm). Cells were illuminated with a 640 nm solid-state laser (diode-pumped, iBeam Smart, Toptica Photonics) and a 488 nm laser (diode-pumped, iBeam Smart, Toptica Photonics). Signals were collected using an Andor iXonEM+ 897 (back-illuminated) EMCCD camera (16 μm pixel size). The following filter sets were used: dichroic filter (ZT405/488/561/640rpc, Chroma Technology GmbH), emission filter (446/523/600/677 nm BrightLine quad-band band-pass filter, Semrock, Rochester), and additional emission filters: ET 700/75 M, Chroma Technology GmbH; ET 525/50 M, Chroma Technology GmbH. For 3D measurements, a cylindrical lens (f = 1,000 mm, Thorlabs) was placed into the optical detection pathway of the microscope.

### 2.7 Two-color 3D SMLM imaging

Simultaneous imaging of the cell’s mitochondria, as well as the activation of platelets, were measured using a two-color beam splitter (OptoSplit II; Cairn Research) with a filter cube for 675 and 525 nm (ET 525/50, H568LPXR, HC 675/67, AHF). Both color channels were projected onto the same camera chip but spatially separated into two non-overlapping spectral channels. To achieve nanometer accuracy, we applied direct stochastic optical reconstruction microscopy (dSTORM) (Heilemann et al., 2008). A cylindrical lens in the optical detection pathway of the microscope introduced astigmatism and allowed for 3D imaging. Axial-depended deformation of the point spread function (PSF) was calibrated using a sample of homogeneous distrusted TetraSpeck™ beads (0.1 µm, Invitrogen) that were moved along the axial axis at defended steps (10 nm) over a range of 200 frames. Since astigmatism depends on the wavelength, both color channels were simultaneously acquired but calibration curves were separately calculated. For imaging, samples were illuminated for 20 ms using both 640 nm and 488 nm lasers at a frame rate of 20 images/s. All illumination protocols were controlled using custom-written acquisition software. For image reconstruction, a sequence of 10,000–20,000 images were recorded, and the single-molecule signals were analyzed using custom-written software (Mayr et al., 2020). The two-color channel regions provided from the calibration images were roughly overlaid. These regions were further used for analysis of each subsequent dSTORM experiment and were the bases for combining the channels. After all single-molecule signals were localized, both channels are combined into one dataset without subpixel chromatic correction. Single-molecule signals with intensities below 500 photons as well as a lateral positional accuracy above 75 nm were discarded. The datasets were drift-corrected using the redundancy cross-correlation (RCC) method (Wang et al., 2014). Due to a small overlap of the Alexa Fluor^©^ 488 emission and the 675/67 nm filter, fluorescent bleed-through had to be corrected. Therefore, random forest classification (Breiman, 2001) to identify single-molecule signals in the Alexa Fluor^©^ 647 channel that were emitted from Alexa Fluor^©^ 488 signals were utilized. A custom-written software enabled the categorization of rendered Alexa Fluor^©^ 647 SMLM images using a brush tool into two classes–certain signals coming from Alexa Fluor^©^ 647 and Alexa Fluor^©^ 488 signals from bleed-through. Features used for the random forest classification included frame number, intensity, background, background error, sigma x and y and a 3 × 3 pixel grid of intensity values from the original image around the signal’s position in both color channels. Random forest classification was then trained and fluorescence data that were not classified as originating from Alexa Fluor^©^ 647 channel were discarded. Finally, the two-color images were rendered using “autumn” and “winter” color maps (adapted from MATLAB^©^) illustrating the axial positions. Each signal was rendered as a symmetrical Gaussian function.

### 2.8 Mitochondria classification

To classify mitochondrial morphology into puncture, rod and network structures, localizations from 3D SMLM had to be converted into 3D volumes. Localizations with lateral and axial positional accuracies of 100 nm and 150 nm, respectively, were filtered. Furthermore, background localizations with insufficient neighbors (density: 0.65 signals/µm³) within a radius of 250 nm were also filtered out. For volume reconstruction, image stacks with a voxel size of 85 nm × 85 nm x 25 nm and 8-bit grayscale color depth were chosen. A lower axial voxel size was used to compensate for higher axial positional accuracies and the maximum depth (1,000 nm) archivable of 3D astigmatism SMLM. Each localization was rendered using an anisotropic 3D Gaussian function, with its sigma dependent on the calculated positional accuracy. Next, the volume data was smoothed using a symmetrical 3D Gaussian filter with a sigma of 1.5 and a window size of 7. These smoothed volumes were then thresholded using a GPU (Cuda, NVIDIA) accelerated adaptive local threshold approach. Herein, for each voxel, the histogram within a box of 11 × 11 × 11 voxels was calculated and the threshold for this voxel was determined by the intermeans (also called iso-data) algorithm (Ridler and Calvard, 1978). Based on the work of Lee et al. (Lee et al., 1994) and the ImageJ plugin “Skeletonize3D” by Ignacio Arganda-Carreras et al., a C++ implementation was created and used to calculate the 3D medial axis or also call “skeleton” from the threshold volumes. The skeleton was analyzed and segmented using a C++ implementation of the ImageJ plugin “AnalyzeSkeleton” from (Arganda-Carreras et al., 2010). Additionally, the volume of each mitochondria segment was reconstructed from the threshold volume using the flood-fill method and volumes with less than 50 detected voxels (originating from mitochondria signals) were discarded. Furthermore, each segment which represents either the skeleton of a single mitochondrion or a cluster of mitochondria was classified into puncture, rod, and networks using random forest classification (parameters: number of branches, number of endpoints, number of junctions, number of slabs, number of triples, number of quadruples, average branch length, maximum branch length, the shortest path, number of voxels, width, height, depth). Training data was generated by manual classification of 2,200 segments. During training, the selected sample size was enough for a reliable classification of unseen mitochondrial segments (see Supplementary Movie S3 for an animation of the mitochondria classification). In a final step, the percentage of voxels in each class was calculated for each EC or cluster of ECs.

### 2.9 Platelet’s volume and density determination

Platelet’s volume and density were determined from 3D SMLM data of CD62P signals. The membrane of individual platelets was approximated via alpha-shapes concave hull algorithm using a MATLAB^©^ script (release 2020b). An alpha value of 1 µm was chosen, and the single-molecule signal densities were calculated from the CD62P signals found within the calculated volume. To determine the number of CD62P signals in the proximity of the platelet’s membrane, the alpha shape hull was shrunken by 60 nm and the number of signals within the shrunken volume was determined. The percentages of CD62P signals in membrane proximity (with a 30 nm radius) were calculated by subtracting the signals within the shrunken hull from the total number of determined CD62P signals (N) and normalized by N.

## 3 Results

### 3.1 Design of the microfluidic platform

**FIGURE 1:**
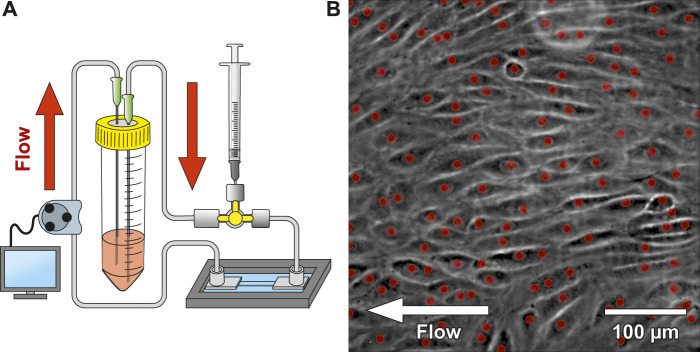
Microfluidic platform design and cell seeding. **(A)** shows a schematic drawing of the microfluidic system. The system was connected to a 3-way stopcock with a septum to allow injecting media containing suspended CD34^+^ endothelial cells (ECs). A peristaltic pump was used to dispense cell culture medium from the reservoir with a variable flow rate. **(B)** shows a phase-contrast image of CD34^+^ adherent ECs within the microfluidic chip after 2 days under constant flow (most ECs aligned with the flow direction). The red dots indicate automatically detected nuclei using a convolutional neuronal network. Number of detected nuclei in the image: 111 on 0.19 mm^2^.

We designed an innovative microfluidic platform to mimic flow conditions within capillaries, under close to physiological conditions to study platelet-endothelial interactions. Figure 1A shows a schematic drawing of the developed microfluidic platform (including chip, tubes, medium reservoir, injection port, and peristaltic pump). The bottom of the chamber consists of a 0.15 mm thick coverslip enabling high NA (NA > 1.4 objective lens, working distance ∼0.3 mm) imaging. A channel (1.5 × 36 mm^2^, 335 µm thick, aspect ratio 4.5, cross area 0.5 mm^2^) was cut into a sandwiched polymer foil with a final channel volume of 17 µL. The Navier-Stokes equation in 3D for rectangular channels according to (Pisapia et al., 2022) was used to calculate the shear stress acting on the ECs. A viscosity value of η=0.875 mPa∙s (99% cell culture medium (RPMI +5% FBS (Poon, 2022)) + 1% whole blood (Roux et al., 2020) to account for the platelets), as well as the given channel length of 34 mm (excluding the inlet size) and a flow rate of 119 μL/min, was used. Additionally, considered was the hydraulic resistance of the 290 cm long tubing. The calculated values for pressure, flow velocity and wall shear stress were 51 Pa, 3.95 mm/s and 62 mPa, respectively. In comparison, typical blood vessel wall shear stress values of 0.1–2 Pa have been reported (Roux et al., 2020). Since we have assumed a viscosity value of platelet concentrate diluted in cell culture medium and not of whole blood, the resulting wall shear stress is only ∼3%, which occurs under physiological conditions.

The microfluidic platform was seeded with CD34^+^ umbilical cord blood cells (CD34^+^, CD45^+^, CD31^+^, KDR^−^, vWF^−^, CD14^−^, ECs; kindly provided by Prof. Grosselet, University of Artois) (Cecchelli et al., 2014), which have previously shown an improved wound healing- and vascular differentiation potential (Fina et al., 1990; Pedroso et al., 2011; Yang et al., 2011; Buchroithner et al., 2021). In our experiments, CD34^+^ umbilical corde blood cell-line that differentiated to ECs (Cecchelli et al., 2014) had tighter intercellular junctions compared to HUVEC/Tert2 (see Supplementary Figure S1). The delivery of ECs into the channel was applied using a 3-way stopcock with a septum and a syringe. A peristaltic pump delivered 595 µL of medium from a reservoir into the channel every 4 h at a flow rate of 119 μL/min. Every 24 h, ECs’ morphology and confluency were observed with phase-contrast imaging. An automated cell counting software was applied to quantify EC proliferation (red dots indicate detected nuclei of Figure 1B). Values around 500 cells/mm^2^ provide a confluency of ∼95–98%. Once confluency was reached, the delay time of the peristaltic pump was gradually decreased (3 h, 2 h, 1 h, 0.5 h and 0.25 h). Simultaneously, the active pump time was increased (5 min, 10 min, 20 min, 30 min) until continuous pumping was reached. ECs were cultured under constant flow conditions at 62 mPa shear stress until on average 65% of them aligned with the flow direction. Figure 1B shows the microfluidic chip seeded with ECs after 2 days of continuous flow, where ECs already align with the flow direction. Live cell platelet interaction experiments under flow conditions were tested on a confluent EC monolayer seeded in the blood vessel chip. We tracked individual platelets (see Supplementary Figure S3A) flowing over the EC monolayer and confirmed platelet adhesion and activation using CD41 and CD62P (see Supplementary Figure S3B), respectively.

### 3.2 Laser treatment of endothelial cells and mitochondrial morphology

**FIGURE 2:**
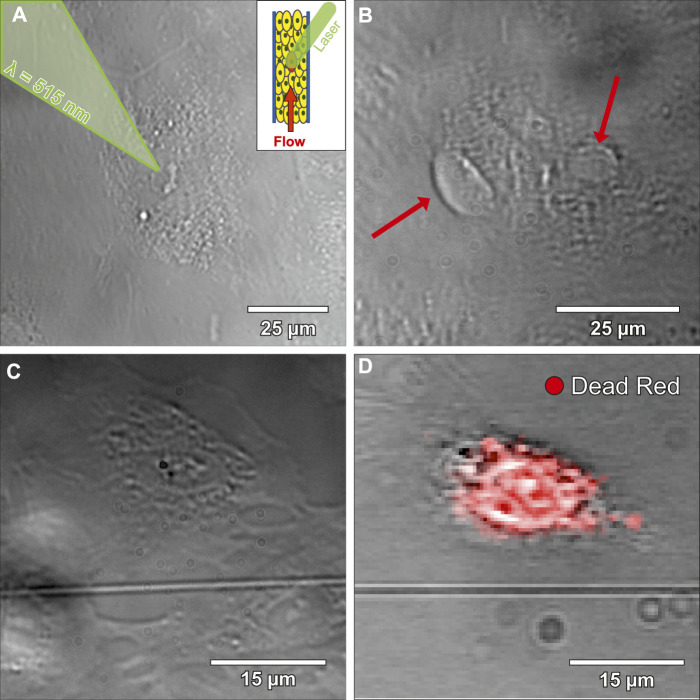
Bright-field microscopy images of laser-treated endothelial cells (ltECs). **(A)** shows an image during the laser treatment process. A femtosecond pulsed laser was used to stress selected cells within the developed microfluidic chip under flow conditions. **(B)** depicts an image of a ltEC forming blebs (red arrows) under flow conditions. **(C)** shows a bright-field microscopy image of a ltEC under static conditions. **(D)** displays an image of the same cell as shown in **(C)** after fixation and overlayed with a LIVE/DEAD™ Red Dead Cell Stain in green, which indicates that ltEC dies within <1 h after laser treatment.

Platelets adhered on a tight and flow-orientated monolayer of perfused ECs (see Supplementary Figure S3B). More precisely, platelets preferably adhered to disrupted intercellular junctions (see Supplementary Figure S1) or to ECs with signs of stress that occurred presumably due to manipulating cells outside the incubator. The 2 cell populations were labelled stress-resistant EC (srEC) and stress-prone EC (spEC) based on platelet adhesion. However, these individually stressed cells were difficult to monitor in the tight cellular layer, via white light microscopy. To gain spatio-temporal control of cellular stress, single EC (ltECs) nuclei were treated with a femtosecond pulsed laser. Experiments (N = 11, three to five ECs treated each) targeting single ECs were carried out using a 515 nm laser (290 fs pulse duration, 1 MHz repetition rate) with a peak power of 1,600 W/μm³ and an air objective lens (50x, NA = 0.42) (Buchegger et al., 2019; Buchegger et al., 2021; Naderer et al., 2024). During laser treatment, ltECs exhibited morphological changes, nucleoplasm leakage, as well as occasionally nuclear bleb formation (Figure 2B). Bleb formation was comparable to experiments with laser treatment of NIH3T3 cells (Wickman et al., 2013). Recent works have also proven that laser irradiation can lead to increased levels of ROS, induced membrane or DNA damage and frequently lead to cell death (Davidson and Duchen, 2007; Tang et al., 2014; Eisner et al., 2018). Cell death of ltECs within <1 h after laser treatment has been confirmed using LIVE/DEAD™ Red Dead Cell Stain (Figures 2C,D).

### 3.3 Platelet binding to endothelial cells

**FIGURE 3:**
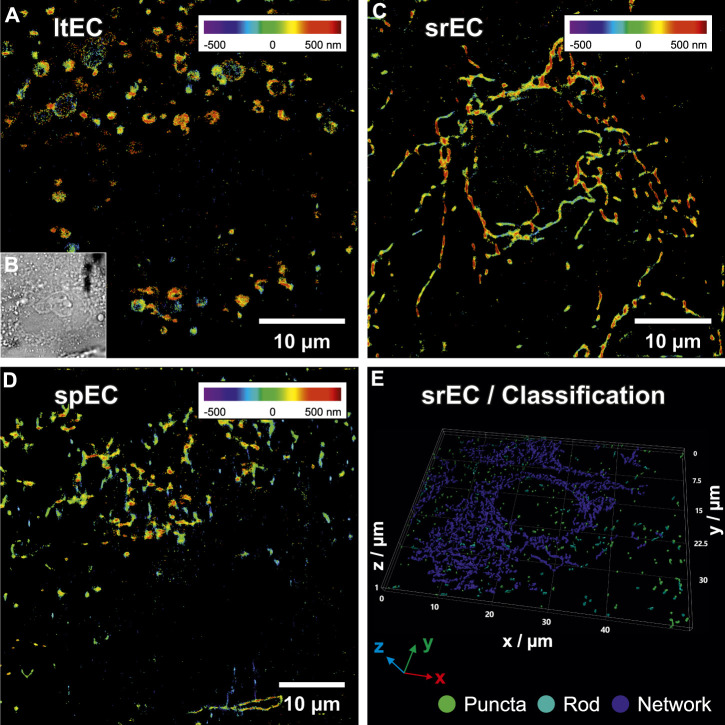
3D single-molecule localization microscopy (SMLM) images of endothelial cells (ECs) mitochondria within the microfluidic chip under flow conditions. **(A)** shows a SMLM rendering of mitochondria (positional accuracy of 46 nm lateral and 53 nm axial) of a laser-treated EC (ltEC) stained with anti-mitochondria antibody conjugated to Alexa Fluor^©^ 488. Axial positions are represented using rainbow colours from violet (below focus) to dark red (above focus). 3D SMLM data were converted into a volume image and each mitochondria segment produced by skeleton analysis was classified using random forest classification. Mitochondria morphology classification of **(A)** resulted in 33% puncta, 23% rods and 44% networks. **(B)** shows a bright-field microscopy image of the same EC as in **(A)**. In **(C)** a reconstructed 3D SMLM image of a stress-resistant EC (srEC) with a continuous mitochondria network (positional accuracy of 51 nm lateral, 80 nm axial, 20% puncta, 20% rods and 60% networks) is displayed. **(D)** depicts a reconstructed 3D SMLM image of a stress-prone EC (spEC) (positional accuracy of 52 nm lateral, 66 nm axial, 29% puncta, 32% rods and 39% networks). **(E)** visualizes a 3D surface reconstruction of a volume rendering from 3D SMLM localizations (positional accuracy of 22 nm lateral and 125 nm axial) of multiple srECs. The results of mitochondrial classification were colour-coded and indicate the categories: puncta (green), rod (cyan) and network (blue) with 8%, 7% and 85% of voxels in each category, respectively (see Supplementary Movie S3 for an animation of the classification procedure).

Subsequently, simultaneous two-color 3D SMLM imaging was used to correlate the stress-classified ECs via mitochondrial organization (green-channel) to the position of platelets and their CD62P distribution (red-channel). The 3D distribution of mitochondria has been used to analyze cellular stress levels of individual ECs within the perfused layer (dead cells confirmed via LIVE/DEAD assay). Three to five individual ECs in the center of the microfluidic chip were selected and subsequently treated using fs-laser pulses (<60 min outside the incubator). The microfluidic chip was placed back into the incubator for 15 min to allow ECs to recover. Subsequently, ECs were fixed and stained for imaging. To analyze the 3D mitochondria morphology, single-molecule positions of anti-mitochondria antibodies were converted into a 3D volume and analyzed. Volumes with a voxel size of 85 nm × 85 nm x 25 nm were rendered from the single-molecule signals as a 3D Gaussian function. These mitochondria volumes determined from the 3D localization positions were subsequently smoothed, thresholded, segmented and skeletonized to quantify their spatial orientation (Arganda-Carreras et al., 2010). Based on the resulting 3D skeletonized image, parameters like segment voxels, number and length of branches were calculated. These parameters were used to classify mitochondria segments into puncta, rod, and network categories using machine learning (random forest classification (Breiman, 2001)). A 3D surface reconstruction (marching cube algorithm (Lorensen and Cline, 1987)) of the classified mitochondria structures is shown in Figure 3E. The colors green, cyan, and blue indicate the categories puncta, rod and network, respectively. The overall results of the classification are presented in Table 1. Based on the distribution of classified voxels in each category, the stress level of single ECs can be determined (Leonard et al., 2015; Zahedi et al., 2018). Large, interconnected mitochondrial networks were observed in srECs (Tang et al., 2014), while puncta and rod-like mitochondria arrangements were highly represented in spECs.

**TABLE 1:** Statistics of cellular mitochondria morphology classification of endothelial cells (ECs) under different conditions. Mitochondria segments of whole and partial ECs were extracted and classified. The percentage of voxels found in each category was then compared to the total number of voxels found within each extracted EC. Median, mean, and standard error were calculated for each experimental condition: static cultivation, dynamic cultivation within the microfluidic chip under flow conditions and laser-treated ECs under dynamic conditions.

State	# ECs	Mean puncta (%)	Median puncta (%)	Mean rods (%)	Median rods (%)	Mean networks (%)	Median networks (%)	Shown in
Static	27	24.9 ± 2.8	24	31.9 ± 3	28	43.1 ± 5.1	48	Figure 3A
Dynamic	16	28.1 ± 3.5	25.5	32.8 ± 3.6	28.5	39.1 ± 5.3	36	Figure 3C
Laser-treated	4	33.5 ± 6.5	31.5	37.5 ± 5	38	29.25 ± 10	37.5	Figure 3D

**FIGURE 4:**
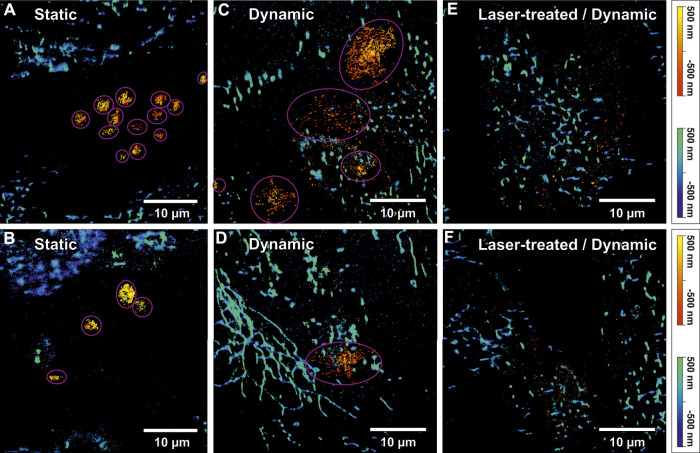
Two-colour 3D single-molecule localization microscopy (SMLM) images of activated platelets on an endothelial cell (EC) layer and mitochondrial networks. **(A)** and **(B)** show SMLM images of activated platelets and mitochondria under static conditions. The pink circles indicate identified single platelets. The “autumn” colour map (sequential increasing shades of red-orange-yellow) represents the axial position of platelets stained using anti-CD62P antibodies (conjugated to Alexa Fluor^©^ 647). The positional accuracy of SMLM signals in **(A)** was 24 nm lateral and 55 nm axial for the red channel and 39 nm lateral and 107 nm axial for the blue channel. For **(B)** a positional accuracy of 31 nm/36 nm lateral and 69 nm/99 nm axial was calculated for the red/blue colour channel, respectively. The “winter” colour map (shades of blue to green) represents the axial positions of mitochondria (anti-Mitochondria marker conjugated to Alexa Fluor^©^488). **(C)** and **(D)** display 3D two-colour SMLM reconstructions of two selected images from a dynamic experiment. Likewise, mitochondria were indicated by “winter” and CD62P on platelets by “autumn” colour maps. Additionally, identified platelets are indicated by pink circles. For **(C)** a positional accuracy of 30 nm/47 nm lateral and 46 nm/75 nm axial and for **(D)** 28 nm/45 nm lateral and 44 nm/75 nm axial were calculated for the red/blue colour channel, respectively. **(E)** and **(F)** show 3D two-colour SMLM reconstructions of a region in proximity of laser-treated ECs (ltECs) under flow conditions. However, the “autumn” colour channel representing activated platelets shows only single-molecule background signals, without any indication of platelets around ltECs. For **(E)** a positional accuracy of 30 nm/49 nm lateral and 46 nm/79 nm axial and for **(F)** 40 nm/49 nm lateral and 63 nm/78 nm axial was calculated for the red/blue colour channel, respectively. It is noteworthy that usually only individua, non-aggregated platelets were bound to the ECs.

Subsequently, the two-color image analysis was used to correlate the EC-mitochondria-distribution to the protein distributions (CD62P) of the locally adherent platelets incubated under various conditions. Experiments were either conducted under static (SC) or dynamic (DC) conditions. In SC experiments, ECs were cultivated and incubated with platelets under static conditions. In DC experiments, ECs were cultivated within the microfluidic system under flow conditions (119 μL/min) and platelets were added into flow. For SMLM imaging, the adhered, fixed platelets were stained with anti-CD62P antibodies conjugated to Alexa Fluor^©^ 647 and mitochondria (platelets, ECs) were stained with anti-mitochondria antibodies conjugated to Alexa Fluor^©^ 488. 3D SMLM images were subsequently corrected for spatial drift (Wang et al., 2014) as well as fluorescent bleed-through (custom software) and the mitochondria morphology was classified. Table 2 shows the voxel classification of mitochondria morphology for each image in Figure 4. Mitochondria of platelets were discarded due to their smaller size during analysis. On average 1.32 ± 1.3 and 1.63 ± 1.4 activated platelets per EC were determined in SC and DC experiments, respectively. We were able to show that CD62P^+^ platelets adhered only in the space between spEC within the EC layer. No CD62P signals could be observed in proximity to ltECs. Moreover, more CD62P^+^ platelets were bound to the EC layer under SC than DC, indicating that shear force affects the binding of platelets to the spEC within the endothelial layer. Since no platelet activation was observed in surroundings of ltECs under DC, we did not test for platelet activation of ltECs under SC.

**TABLE 2:** Voxel classification results of the cellular mitochondria segments analyzed from all ECs in each image of Figure 4 under different conditions (static, dynamic and laser-treated ECs). The percentage of voxels found in each class compared to the total number of voxels is presented in the columns “puncta”, “rod” and “networks”. The number of identified platelets for each image is presented in the column “# Platelets”.

State	Puncta (%)	Rod (%)	Networks (%)	# Platelets	Shown in
Static	31	30	39	13	Figure 4A
	7	18	75	4	Figure 4B
Dynamic	41	32	27	5	Figure 4C
	14	15	71	1	Figure 4D
Laser-treated	22	34	44	0	Figure 4E
	26	27	47	0	Figure 4F

### 3.4 P-selectin clustering on adherent platelets

3D single-molecule fluorescence signals of the labelled CD62P were used to approximate the volume of individual platelets via the alpha-shapes concave hull algorithm. The number of identified platelets in each image in Figure 4 is presented in Table 2. A total of thirteen and four round-shaped platelets are displayed in Figures 4A,B, respectively. Figure 4C shows four single platelets spread between spECs. Platelets under SC (N = 31 platelets) showed a spherical shape with certain aggregate formation (Figures 4A,B) presumably in the intercellular space of the EC layer. Since platelets have to withstand shear force while interacting with spECs, larger activated platelets were observed under DC compared to SC. Only ∼27% of the platelets activated under DC (total N = 37 platelets) were in a similar volume range compared to the platelets activated under SC. We observed on average 6 times larger volumes of CD62P^+^ platelets adhering to spECs under DC (compared to SC, see Table 3). In addition to the volumes, the CD62P densities in the platelet membranes were also determined and compared using 2CALM analysis (2-sample Comparative Analysis of 3D Localization Microscopy Data (Mayr et al., 2020)) regarding their differences in density or the shape of the clusters formed. Using the calculated alpha-shapes, we show that for both activation conditions ∼3.8/∼6.87 times more CD62P proteins are present in the platelet membrane (first 60 nm layer) compared to other layers in activated platelets under SC and DC (see Supplementary Figure S5). In total, the inner layers harbor ∼57% and ∼31% of CD62P in activated platelets under SC and DC respectively; the contributing proteins were stored in the alpha-granules. Here we show that the CD62P reservoirs have been more depleted in the dynamically activated platelets. In total numbers ∼20,000 and ∼3,000 CD62P localizations have been observed on the membrane activated platelets under DC and SC respectively. A more precise analysis of the CD62P protein surface distribution indicated that, ∼62% for activated platelets under DC (CD62P in the upper membrane/bottom membrane: 5,586 ± 170/14,524 ± 338 for N = 37 platelets) and 56% for activated platelets under SC (CD62P in the upper membrane/bottom membrane: 863 ± 33/1,979 ± 213 for N = 31 platelets; see Table. 3) were localized in the bottom half of the platelets, in the EC membrane proximity.

**TABLE 3:** Statistical comparison of activated platelet volumes and densities from 3D single-molecule signals of CD62P under static and dynamic conditions.

State	# Platelets	Mean signal density of CD62P in the Mambrane	Median platelet volume	Mean platelet volume	Platelet volume 25% quantile	Platelet volume 75% quantile
Static	31	734 ± 261 signals/µm³	1.4 µm³	1.6 ± 0.22 µm³	0.67 µm³	1.9 µm³
Dynamic	37	808.5 ± 553.1 signals/µm³	6.6 µm³	9.4 ± 1.5 µm³	3 µm³	12 µm³

The variation of total number of localized CD62P within the activated platelets under SC and DC might be introduced by the reduced accessibility of the antibodies to the CD62P in granules and capability of shading. Thus, we observe a higher CD62P density at the EC/platelet interface relative to the upper half of the cell; this tendency decreases for SC. Next, we compared the clustering on top and bottom (EC/platelet interface) of the activated platelets under SC/DC. 2CALM analysis, have been performed to quantify and compare the CD62p clustering (platelet’s upper membrane/bottom membrane), under DC. The pairwise 2CALM comparison of the CD62P clusters shows a similarity of the clusters in the upper membrane (N = 7; with sufficient CD62P density for cluster analysis out of N = 31) to the lower membrane (N = 25) upon dynamic activation for ∼85% of the compared pairs (see Supplementary Figure S6A). For activated platelets under SC the CD62P densities are not sufficient high for a statistically meaningful comparative cluster analysis. Solely three statically (out of N = 31 analyzed) activated platelets had sufficient CD62P molecules at the EC/platelet interface (bottom membrane) for a statistical comparison of the CD62P clustering to the dynamically activated platelets (N = 25). Nevertheless, the pairwise comparison of the CD62P clusters between the bottom membranes of activated platelets under SC and DC show dissimilarity for most of the compared pairs (see Supplementary Figure S6B). Moreover no platelets were adhering to ltECs under DC as exemplified by Figures 4E,F. The inability of platelets to adhere indicates that these ltECs were unable to express adhesion molecules to facilitate platelet binding, presumably due to nuclear damage.

## 4 Summary

Single-molecule localization microscopy (SMLM) enables imaging of subcellular structures and proteins with nanometer precision to study platelet endothelial interaction under static and dynamic conditions. Next to image reconstruction, SMLM data can be directly used to Figures 4E,F relevant information in a post-processing step. The machine learning supported analysis enabled the correlation of 3D localization positions/densities of activation markers with the mitochondria morphology of stressed/un-stressed ECs. Here we can show that applying two levels of stress to ECs, one that approximates the physiological conditions of hypoxia under cultivation and handling, and the other induced by laser beam damage, we were able to quantify the cellular stress based on mitochondrial morphology. Simultaneous two-color 3D SMLM was capable to generate additional value for non-diffraction limited objects like mitochondria. For data analysis: filtering of outliers, background signals and residual fluorescent bleed-through was successfully applied to the localized positions. 3D volume reconstruction from SMLM mitochondria localizations allowed us to use well-established algorithms for mitochondria morphology analysis while still preserving the localization data.

The higher resolution of 3D mitochondria images helped to segment the mitochondrial network and reduced the complexity of pre-processing steps compared to example confocal volume analysis. The added value of this work is the simultaneously acquired two-color SMLM (mitochondria and CD62P) data, for volume and density comparison of activated platelets under static and dynamic conditions. In the future, our platform could be useful to study complex medical conditions such as platelet binding to the pre-treated endothelium, platelet to platelet- or platelet to neutrophil binding, thrombus formation (Tsai et al., 2012), the result of pathogens and toxins on platelet adhesion (Fulda et al., 2010) as well as pharmacological effects in the interaction of platelets with the endothelium (Bieberich et al., 2021) at the single-cell level. Additional variables such as the effect of cellular stress could be studied under controlled conditions. This new technology will help clinicians and researchers in their task to omit animal studies when searching for adequate and precise technologies to investigate dynamic platelet interaction with the endothelium under physiological and pathophysiological conditions.

## Data Availability

The datasets presented in this study can be found in online repositories. The names of the repository/repositories and accession number(s) can be found below: https://github.com/CURTLab/NanoMito3D-Platform.

## Abbreviations

SMLM: single molecule localization microscopy, ltECs: laser-treated endothelia cells, srECs: stress-resistant endothelia cells, spECs: naturally stress-prone endothelia cells, dSTORM: direct stochastic optical reconstruction microscopy.
