# Supplementary material for: Single molecule studies of dynamic platelet interactions with endothelial cells
Source: Front Bioeng Biotechnol. 2024 Apr 3;12:1372807. doi: 10.3389/fbioe.2024.1372807 (PMC11025363; doi:10.3389/fbioe.2024.1372807)
Supplement: Supplementary file 5 [file DataSheet1.PDF]

Supplementary Material for “Single Molecule Studies of Dynamic Platelet Interactions with Endothelial Cells”

1 Supplementary Text

1.1 Platelet tracking protocol

Perfusion experiments were conducted under flow conditions using the microfluidic system (as described in the main text), where the rolling potential of platelets on a previously perfused confluent layer of $CD34^+$ ECs were monitored using live cell bright-field imaging. For that purpose, human platelets from concentrates obtained by single platelet apheresis were diluted with ECM-5 and perfused over the EC layer. Due to a limited frame rate, tracking platelets at a flow rate of 119 µL/min was unfeasible in our system. Thus, platelets were propelled using hydrostatic force generated by filling only one tube connector with the platelet solution. The hydrostatic force allowed a continuous flow of platelets for 10 minutes. Suppl. Figure S3a shows a bright-field image with platelets moving over an EC layer inside the microfluidic chip. Image capturing trajectories and cell interaction of the inserted platelets were acquired at 0.48 Hz (suppl. Movie S1). For two-color immunostaining, P-selectin (CD62P, 100 µg/mL, BioLegend) conjugated Alexa Fluor© 647 was used to stain activated platelets and Integrin α-IIb (CD41, A4-309-T100, Exbio) conjugated Alexa Fluor© 488 to visualize all platelets.

Intensive binding of platelets to the EC layer as well as the tendency to form aggregates was observed under flow conditions (suppl. Figure S3b and suppl. Movie S2). The activation of platelets has been observed by expression of adhesion molecule CD62P (suppl. Figure S1b). Only 66% of adherent $CD41^+$ platelets showed co-expression of CD62P (we did not observe the secretion of soluble P-selectin under these experimental conditions).

1.2 Tracking of platelets under flow conditions

For platelet tracking, the microfluidic system was prepared as described in the main text. The medium within the bioreactor reservoir was then exchanged with a mixture of 10% human platelet concentrate and ECM-5. The tubing of the microfluidic chip was removed, and the chip was then placed on an Olympus IX81 microscope with a 100x oil-immersion objective lens (UApo N 100x/1.49 NA, Olympus). The flow was generated using the hydrostatic flow by only filling a single tube connector (83.2 µL) with the platelet solution. A total of 1000 bright field images at a frame rate of 0.48 Hz and an illumination time of 100 ms were acquired. Platelet positions of the first 50 frames were manually marked. Platelet trajectories were calculated using the python toolbox trackpy [1]. Trackpy provides a method ‘compute_drift’ which was used to calculate the mean flow speed platelets under flow conditions generated by hydrostatic forces.

1.3 References

[1] D. B. Allan, T. Caswell, N. C. Keim, C. M. van der Wel, and R. W. Verweij, “trackpy.” p. v0.5.0, 2021.

[2] S. Mayr, F. Hauser, S. Puthukodan, M. Axmann, J. Göhring, and J. Jacak, “Statistical analysis of

3D localisation microscopy images for quantification of membrane protein distributions in a platelet clot model," *PLOS Comput. Biol.*, vol. 16, no. 6, p. e1007902, Jun. 2020.

2 Supplementary Figures

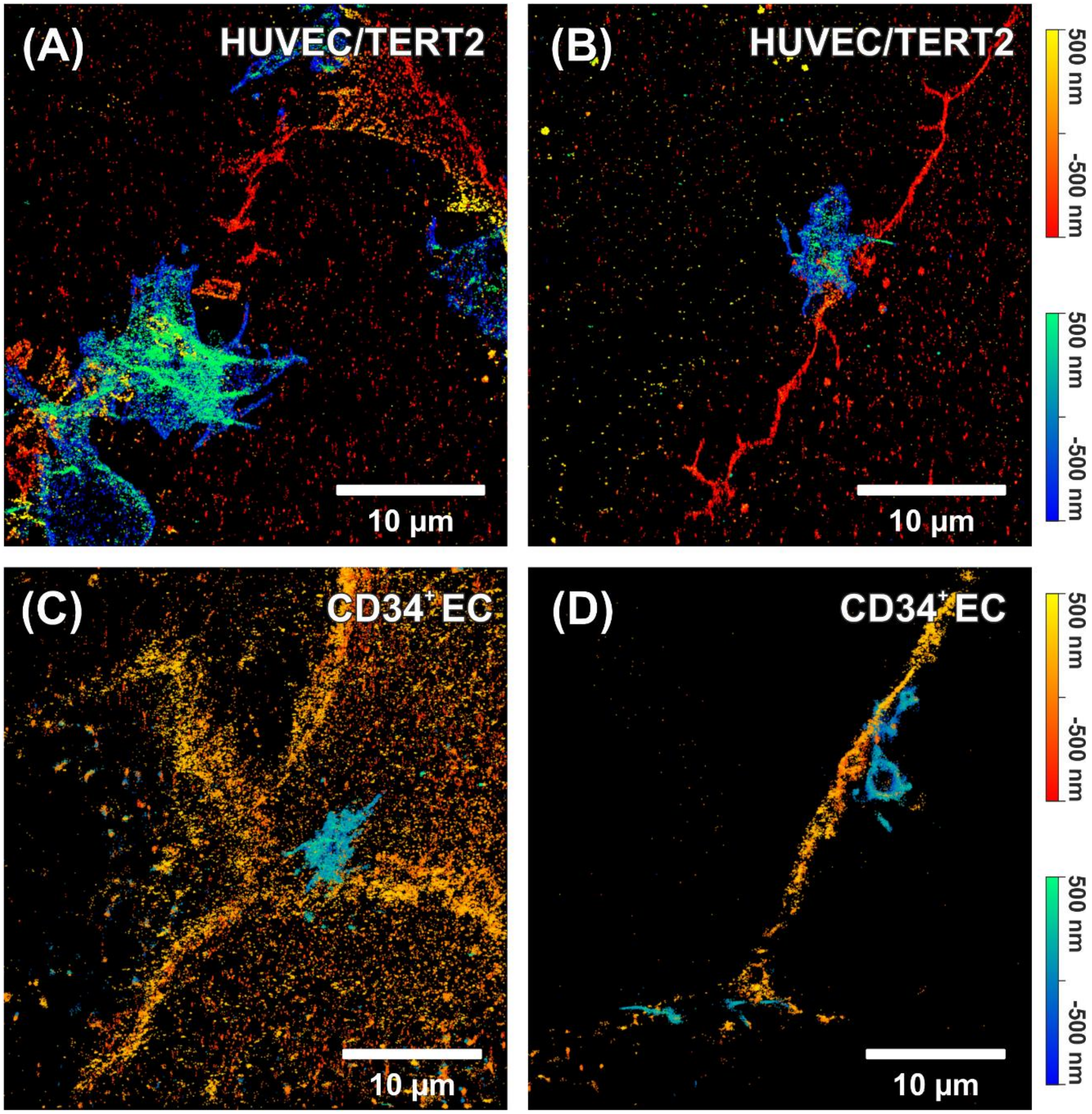

Supplementary Figure S1: Simultaneous two-colour 3D single molecule localization microscopy images of adherence junctions of endothelial cells (ECs) and platelets on an endothelial monolayer. The "autumn" colour map visualizes the axial positions (yellow above focus, red below focus) of adherence junctions labelled with anti-CD144 (VE-cadherin) conjugated to Alexa Fluor© 647. The "winter" colour map visualizes the axial positions (green above focus, blue below focus) of the surface protein CD41 with anti-CD41 conjugated to Alexa Fluor© 488. Colour channels are pixelwise aligned and single molecules were rendered with a width of 30 nm. a), b) shows a HUVEC/TERT2 and c), d)

$CD34^+$ endothelial cell, both cultivated within a microfluidic chip under flow conditions (flow rate 119 µL/min) for 48h. Platelets were incubated under flow conditions for 15 min at 37°C.

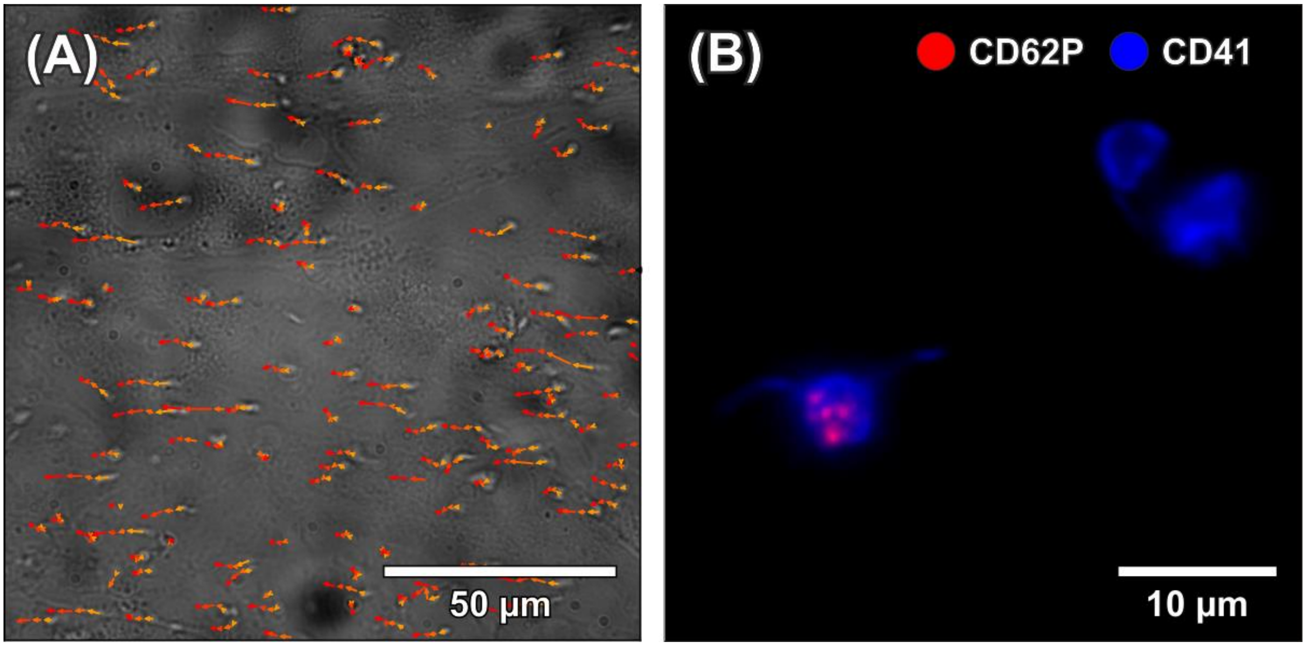

Supplementary Figure S2: Platelet test experiments under flow conditions. a) displays a bright-field microscopy image of an EC monolayer and platelets flowing above. The arrows indicate the movement of single platelets over 4 frames (start: orange, end: red). The positions of platelets were tracked using trackpy [1]. In b) an overlay of two fluorescent images of platelets on $CD34^+$ ECs under flow conditions co-stained with anti-CD41 antibodies conjugated to Alexa Fluor© 488 (blue) and anti-CD62P antibodies conjugated to Alexa Fluor© 647 (red) is shown.

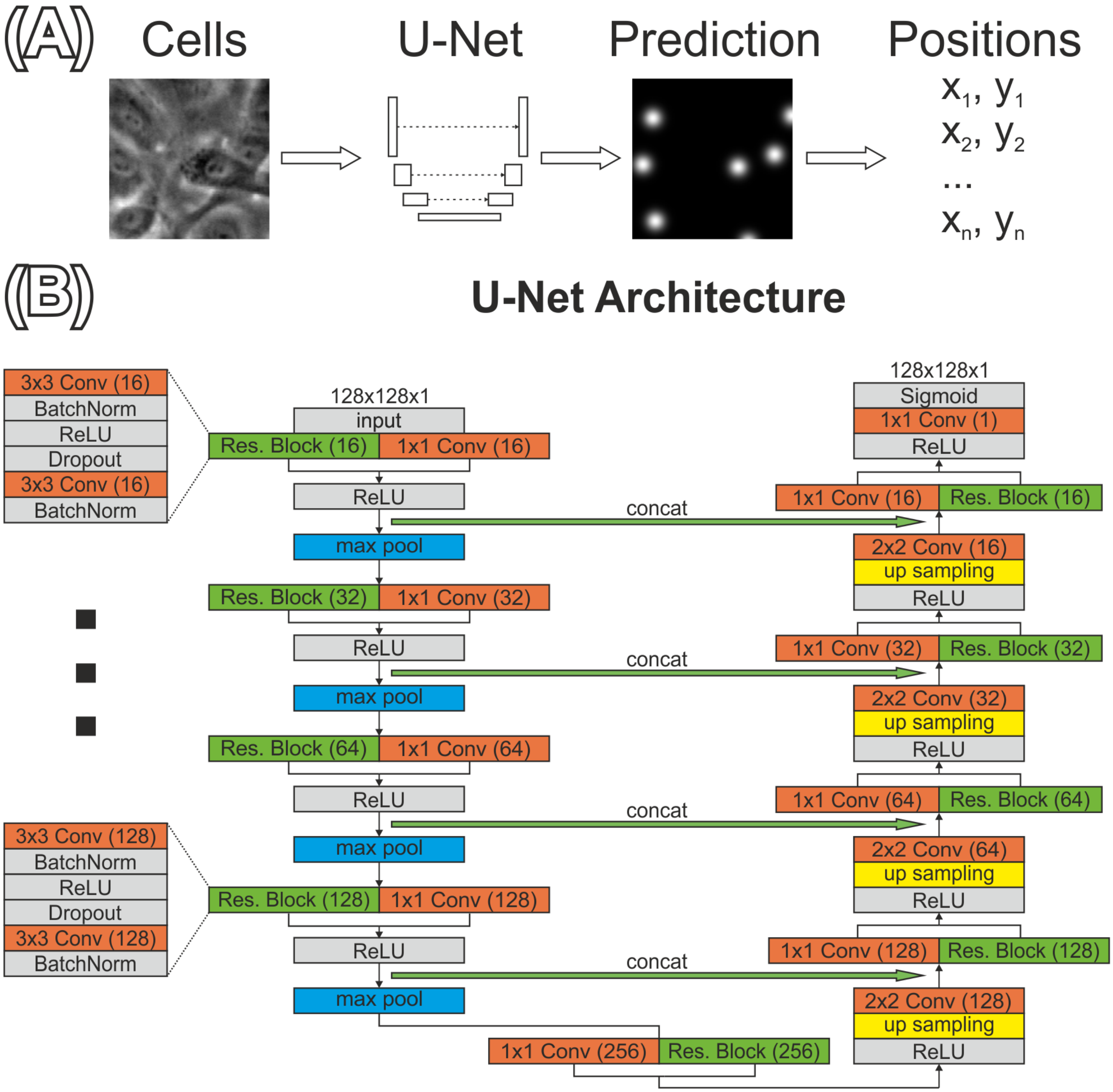

Supplementary Figure S3: General approach for cell nuclei segmentation and UNet architecture. a) The network receives a 128x128 sub image of endothelial cells imaged using phase contrast microscopy. Its output is the predicted cell nuclei as 2D symmetrical Gaussian function (sigma 5 pixels). Based on these predicted Gaussian functions positions are calculated via non-maximum suppression. b) shows the network architecture which is a combination of residual neuronal networks and U-Net. The network consists of 2 030 817 trainable parameters.

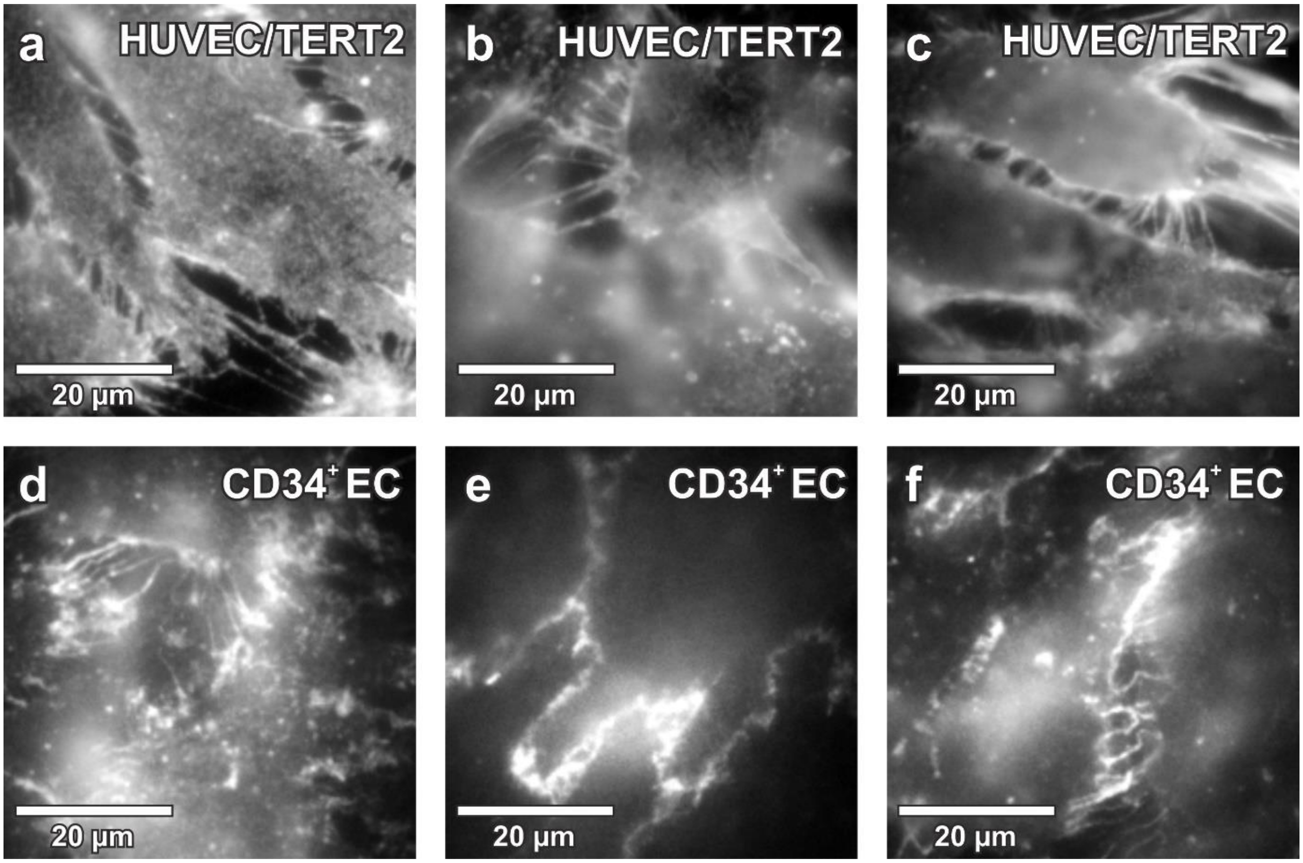

Supplementary Figure S4: Fluorescent images of the endothelial cell-cell adhesion protein PECAM-1 stained with anti-CD31 conjugated to Alexa Fluor© 647. a)-c) shows a HUVEC/TERT2 and d)-f) shows $CD34^+$ primary human endothelial cell, both cultivated within a microfluidic chip under flow conditions (flow rate 119 µL/min) for 48h.

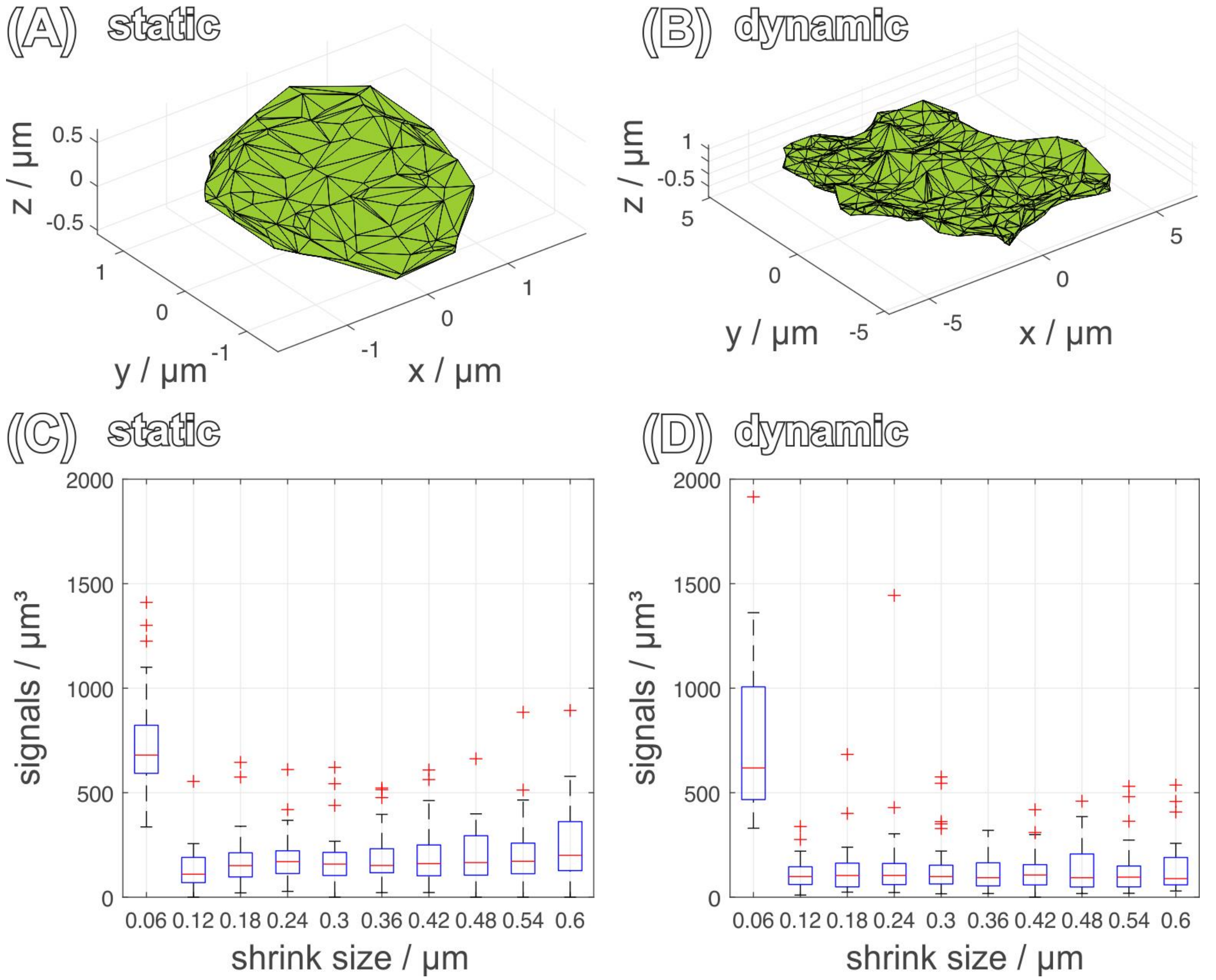

Supplementary Figure S5: Analysis of platelet volumes and densities under static and dynamic flow conditions. Volumes were calculated from CD62P distributions of 3D single molecule localization microscopy (SMLM) images using MATLAB's alpha-shapes concave hull algorithm. Firstly, single platelets were manually cut out of the SMLM images based on their morphology. Then, the membrane of each platelet was approximated based on the CD62P distribution (using alpha shapes with an alpha value of 1 µm). The determined hull was then shrunken in 60 nm steps. At each step, the volume and number of CD62P signals within this volume was calculated. The density was calculated by first subtracting the determined volume/signals from the step before and dividing the signals by the volume. a) and b) show a 3D reconstruction of platelet's concave hull approximated by the CD62P distribution under static and dynamic flow conditions, respectively. c) shows a boxplot of stepwise hull shrinkage and density calculations of CD62P for each individual platelet (N platelets = 34) under static. Like c), d) shows a boxplot of densities for the CD62P distribution of each individual platelet (N platelets = 49) under dynamic flow conditions at several shrinkage steps.

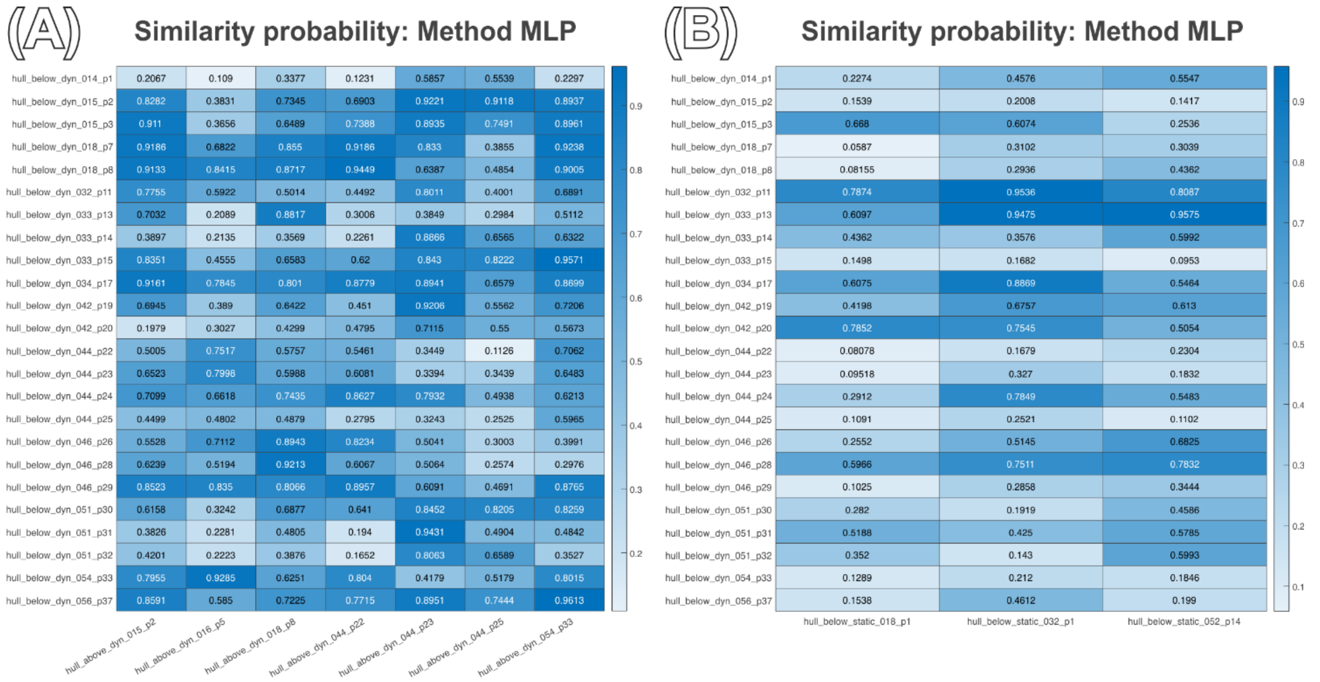

Supplementary Figure S6: Pairwise comparison of CD62P membrane. a) Results on direct pairwise comparison of the CD62p clustering between platelet’s upper membrane/bottom membrane under dynamic activation conditions. 7 distributions of CD62P at the upper platelet membrane (n>200 CD62P’s) and 25 distributions of CD62P at the bottom platelet membrane of dynamically activated platelets were compared using the 2CALM software. The represented data shows multilayer perceptron (MLP) neural network, a classifier for sample similarity combining 11 statistical parameters into 2 neuronal outputs describing the similarity [2]. b) Similar analysis as in a) has been performed for pairwise comparison of the bottom membranes of statically, dynamically activated platelets. 3 statically activated CD62P platelet distributions (n>200 CD62P localisations) were compared to 25 dynamically activated platelet.

3 Supplementary Movies

Supplementary Movie S1: Bright-field microscopy image sequence of platelet added to the flow of the developed microfluidic chip seeded with a HUVEC/TERT2 monolayer. Images were acquired at 0.48 Hz and platelets were propelled by hydrostatic force by filling only one tube connector with 1:10 diluted platelet concentrate with HUVEC/Tert2 medium. The platelet positions of the first 30 frames were analysed using trackpy and a mean platelet velocity of 5.8 µm/s was calculated.

Supplementary Movie S2: Fluorescence image sequence of platelet flowing above a HUVEC/TERT2 monolayer within the developed microfluidic chip. The image sequence was acquired at 0.1 Hz. Platelets were propelled by hydrostatic force by filling only one tube connector with 1:10 diluted platelet concentrate with HUVEC/Tert2 medium. Additionally, the medium is stained using 1:100 diluted Anit-CD41 antibodies (conjugated to Alexa Fluor 488).

Supplementary Movie S3: Animation showing the classification of mitochondria: The mitochondrial channel of a two-colour 3D single-molecule localization microscopy dataset is rendered as a 3D volume, thresholded and skeletonized. In a final step, machine learning is used to classify the mitochondrial segments into the categories: dots (green), rods (cyan) and network (blue).
